# Supplementary material for: p-Coumaric Acid Enhances Hypothalamic Leptin Signaling and Glucose Homeostasis in Mice via Differential Effects on AMPK Activation
Source: Int J Mol Sci. 2021 Jan 31;22(3):1431. doi: 10.3390/ijms22031431 (PMC7867021; doi:10.3390/ijms22031431)

Supplementary information

p-Coumaric Acid Enhances Hypothalamic Leptin Signaling and Glucose Homeostasis in Mice via Differential Effects on AMPK Activation

Linh V. Nguyen [1], Khoa D.A Nguyen [1], Chi-Thanh Ma [2], Quoc-Thai Nguyen [2], Huong TH. Nguyen [3], Dong-Joo Yang [4], Trung Le Tran [4], Ki Woo Kim [4,*] and Khanh V. Doan [1,3,*]

[1] School of Medicine, Tan Tao University, Duc Hoa, Long An, 850000, Vietnam

[2] Faculty of Pharmacy, University of Medicine and Pharmacy, Ho Chi Minh, 700000, Vietnam

[3] School of Pharmacy, Van Lang University, Ho Chi Minh, 700000, Vietnam

[4] Departments of Oral Biology and Applied Biological Science, BK21 Plus, Yonsei University College of Dentistry, Seoul, 03722, Korea

*Corresponding authors: Email: kiwoo-kim@yuhs.ac (K.W.K); doankhanh.pharm@gmail.com (K.V.D.)

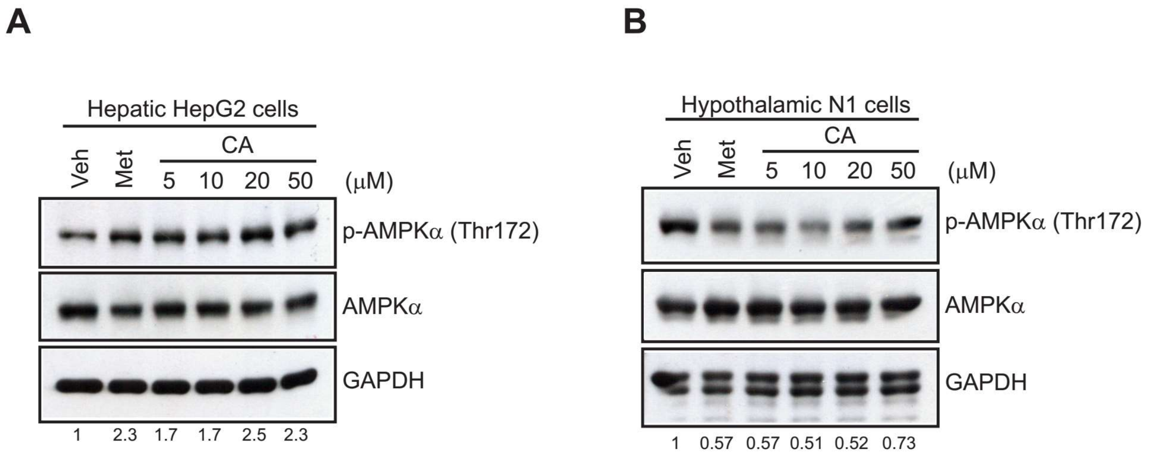

Figure S1. Dose-dependent *in vitro* effects of *p*-coumaric acid (CA) treatment on AMPK activation. (**A**) Immunoblots showing dose-dependent effect of CA treatment on AMPK activation in HepG2 cells. (**B**) Immunoblots showing dose-dependent effect of CA treatment on AMPK activation in N1 cells. The digitals at the bottom indicated p-AMPK/AMPK ratios normalized to corresponding GAPDH levels. Veh: vehicle, Met: metformin 2 mM.

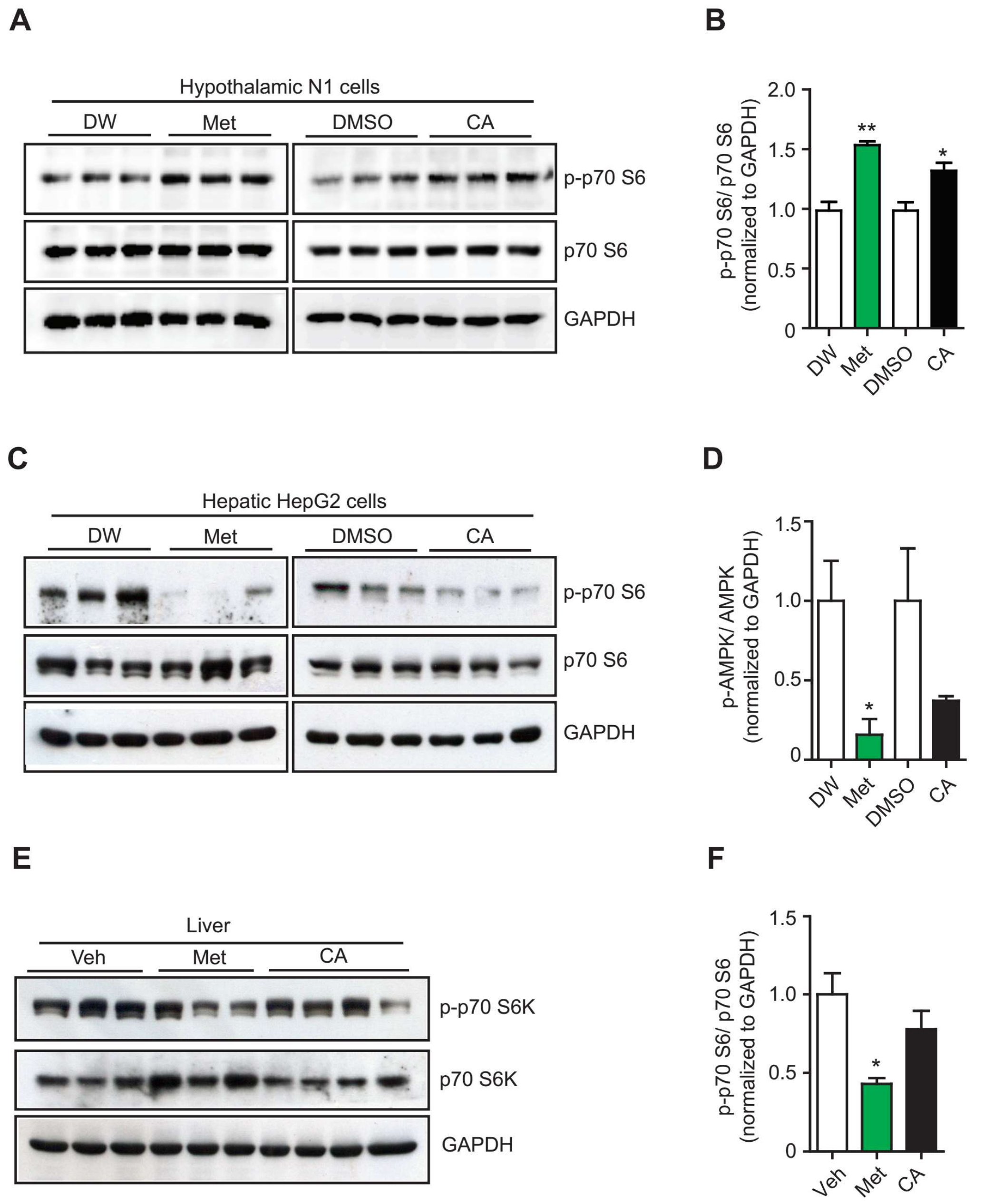

Figure S2. Effects of *p*-coumaric acid (CA) treatment on S6 kinase activity. Immunoblots (**A**) and graph (**B**) showing levels of p-p70S6 and p70S6 in hypothalamic N1 cells treated with metformin (2 mM) and CA (10 μM). Immunoblots (**C**) and graph (**D**) showing levels of p-p70S6 and p70S6 in hepatic HepG2 cells treated with metformin (2 mM) and CA (20 μM). Immunoblots (**E**) and graph (**F**) showing levels of p-p70S6 and p70S6 in livers of mice orally treated with 200 mg/kg of metformin and CA. The results are expressed as mean ± SEM. One-way ANOVA with Tukey's post-hoc tests for comparison of multiple groups or Student's t-test. $^{*}P < 0.05$, $^{**}P < 0.01$.

Original blots

Figure 1A

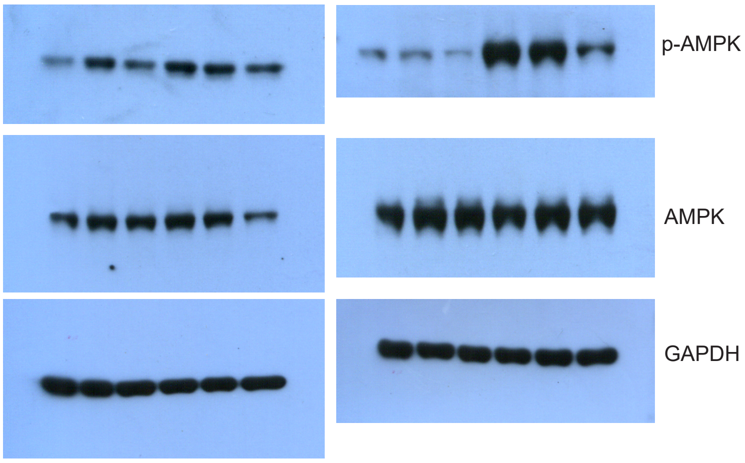

Figure 2A

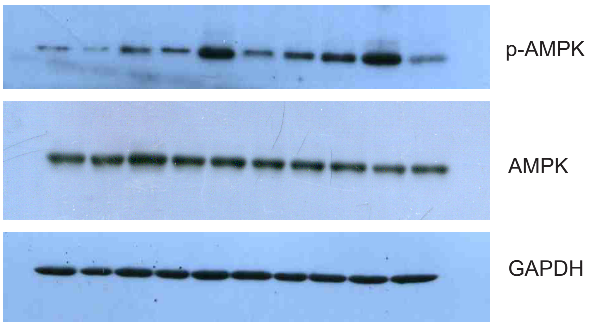

Figure 2C

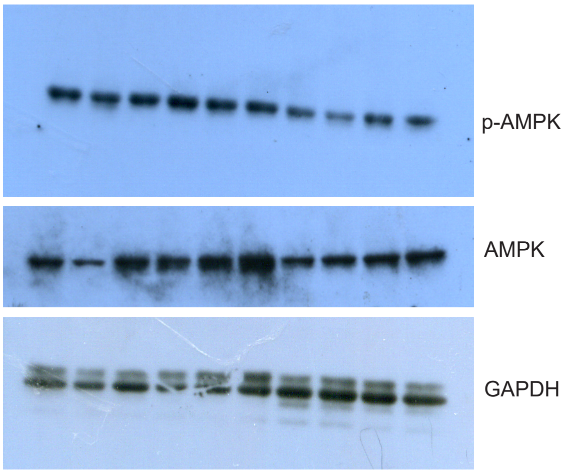

Figure 1C

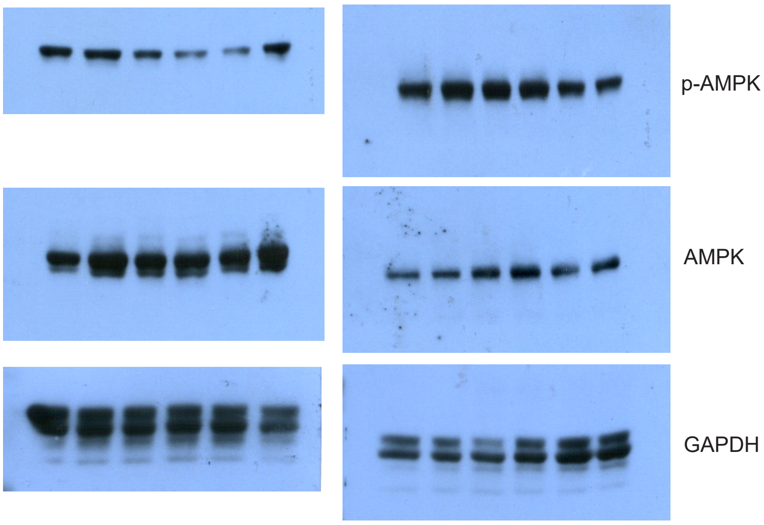

Figure 2E

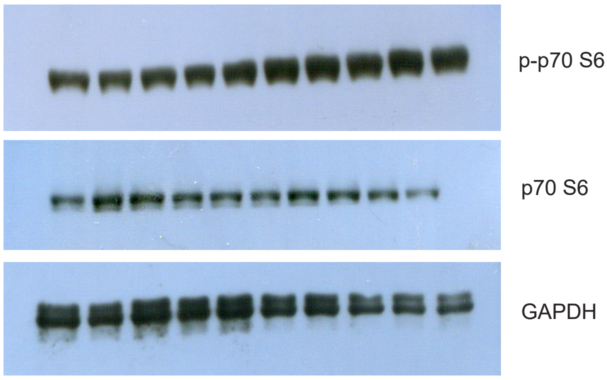

Figure 3B

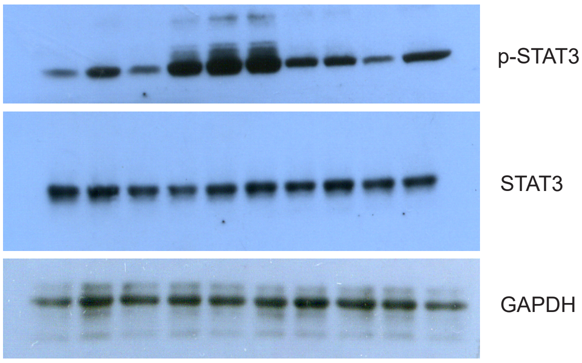

Figure S1A

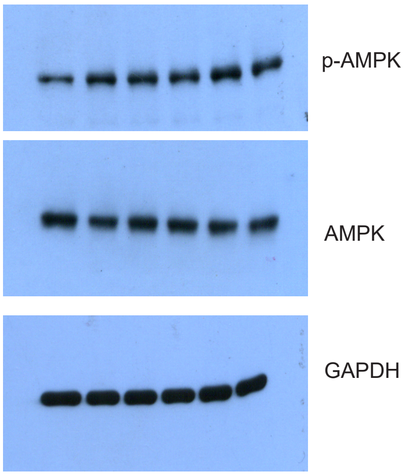

Figure S1B

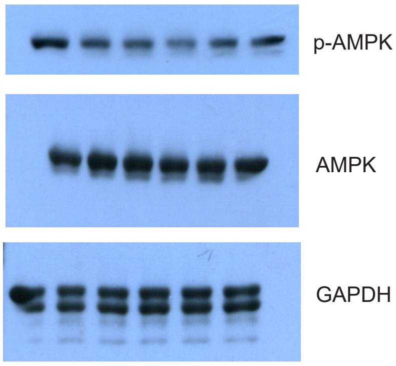

Figure S2A

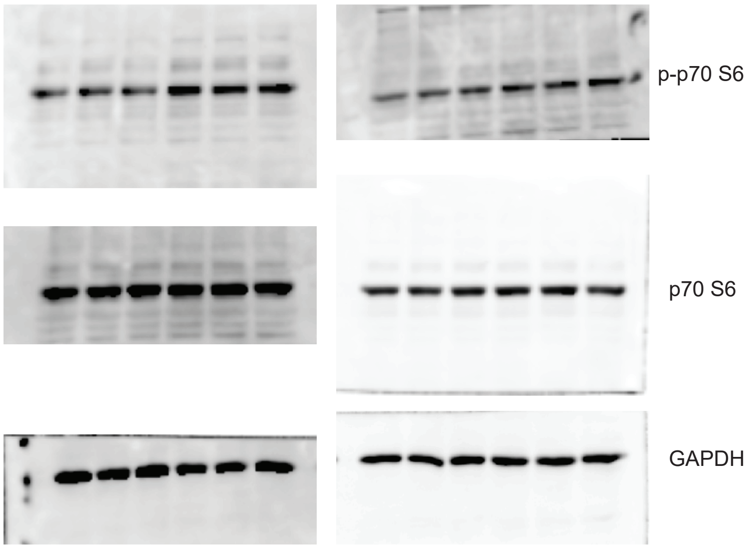

Figure S2E

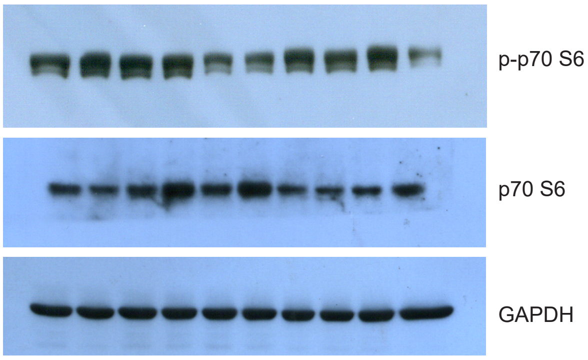

Figure S2C

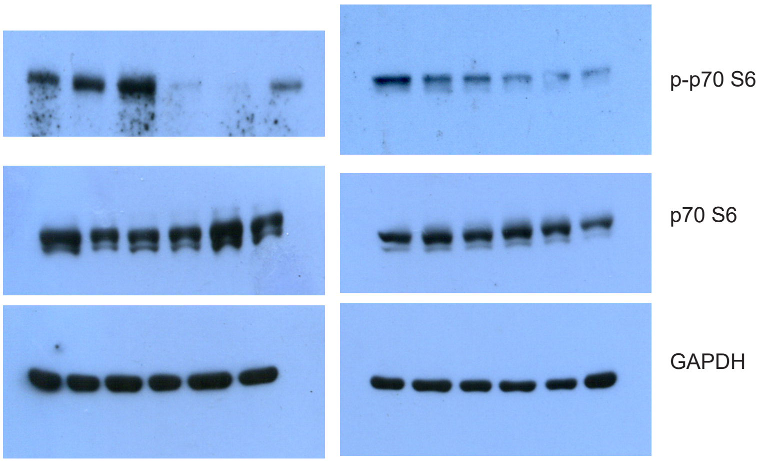

Supplement: Supplementary file 1 [file ijms-22-01431-s001.pdf]
